# Studies on metal–organic framework (MOF) nanomedicine preparations of sildenafil for the future treatment of pulmonary arterial hypertension

**DOI:** 10.1038/s41598-021-83423-6

**Published:** 2021-02-22

**Authors:** Nura A. Mohamed, Haissam Abou-Saleh, Yu Kameno, Isra Marei, Gilberto de Nucci, Blerina Ahmetaj-Shala, Fisnik Shala, Nicholas S. Kirkby, Lewis Jennings, Dana E. Al-Ansari, Robert P. Davies, Paul D. Lickiss, Jane A. Mitchell

**Affiliations:** 1 Department of Biological and Environmental Sciences, College of Arts and Sciences, Qatar University, Doha, Qatar; 2 Department of Chemistry, White City Campus, Imperial College, London, UK; 3 Department of Cardiothoracic Pharmacology, National Heart and Lung Institute, Imperial College, London, UK; 4 Qatar Foundation Research and Development Division, Doha, Qatar; 5 Department of Pharmacology, Faculty of Medical Sciences, State University of Campinas (UNICAMP), Campinas, SP Brazil; 6 Department of Pharmacology, Institute of Biomedical Sciences, University of São Paulo, São Paulo, SP Brazil; 7 Biomedical Research Center, Qatar University, Doha, Qatar

**Keywords:** Nanoscience and technology, Nanomedicine, Drug delivery

## Abstract

Pulmonary arterial hypertension (PAH) is an incurable disease, although symptoms are treated with a range of dilator drugs. Despite their clinical benefits, these drugs are limited by systemic side-effects. It is, therefore, increasingly recognised that using controlled drug-release nanoformulation, with future modifications for targeted drug delivery, may overcome these limitations. This study presents the first evaluation of a promising nanoformulation (highly porous iron-based metal–organic framework (MOF); nanoMIL-89) as a carrier for the PAH-drug sildenafil, which we have previously shown to be relatively non-toxic in vitro and well-tolerated in vivo. In this study, nanoMIL-89 was prepared and charged with a payload of sildenafil (generating Sil@nanoMIL-89). Sildenafil release was measured by Enzyme-Linked Immunosorbent Assay (ELISA), and its effect on cell viability and dilator function in mouse aorta were assessed. Results showed that Sil@nanoMIL-89 released sildenafil over 6 h, followed by a more sustained release over 72 h. Sil@nanoMIL-89 showed no significant toxicity in human blood outgrowth endothelial cells for concentrations up to100µg/ml; however, it reduced the viability of the human pulmonary artery smooth muscle cells (HPASMCs) at concentrations > 3 µg/ml without inducing cellular cytotoxicity. Finally, Sil@nanoMIL-89 induced vasodilation of mouse aorta after a lag phase of 2–4 h. To our knowledge, this study represents the first demonstration of a novel nanoformulation displaying delayed drug release corresponding to vasodilator activity. Further pharmacological assessment of our nanoformulation, including in PAH models, is required and constitutes the subject of ongoing investigations.

## Introduction

Pulmonary arterial hypertension (PAH) is a devastating disease within which pulmonary arteries constrict and remodel, resulting in elevated pulmonary artery pressure and increased workload on the right side of the heart. This ultimately leads to right heart failure and premature death. Whilst there is no cure for PAH, there are four classes of vasodilator drugs currently used to help slow disease progression. These drugs include; (i) phosphodiesterase type 5 inhibitors, such as sildenafil^1^, (ii) soluble guanylate cyclase activator drugs, such as riociguat^2^, (iii) endothelin-1 receptor antagonists^3^, such as bosentan^4^, and (iv) drugs acting on the prostacyclin pathway, such as the synthetic prostacyclin (epoprostenol)^5^, prostacyclin analogs such as treprostinil sodium, iloprost, beraprost^6,7^ and finally the selexipag^8^ which is a selective prostacyclin IP receptor agonists. Whilst these drugs show efficacy against PAH, they have limited pharmacokinetics and affect the systemic circulation, which ultimately limits the dose of drug that can be used. As such, we^9–11^ and others^12–14^ have suggested that PAH is a disease that would benefit from the application of controlled drug release, with the future possibility of introducing targeted drug delivery strategies, using a nanomedicine approach. Although this idea is relatively novel, a limited number of studies are emerging describing nanomedicine formulations suitable for application in PAH. These include (i) a liposome-conjugate that combines the Rho-kinase inhibitor and vasodilator drug fasudil with the nitric oxide (NO) donor DETA NONOate^15^, (ii) a liposome-encapsulated Iloprost nano-formulation^16^ and from our group, a polymeric NO-releasing nanoparticle^11^. Unfortunately, liposomes and polymers as nanoformulation platforms have limitations, including (i) difficulty of in vivo imaging without further chemical modification, (ii) low solubility window, (iii) difficulty of drug fusion and encapsulation, (iv) high production cost, and (v) difficulty in maintaining stability and bioactivity of drugs during the conjugation process^17,18^. We have previously highlighted the potential of nanoscale metal–organic frameworks (MOFs; nanoMOFs) as carriers for PAH drugs. In this regard, MOFs have several advantages as drug delivery platforms including (i) being biocompatible and biodegradable, (ii) high thermal, chemical, and mechanical stability, (iii) control over nanoparticle size, (iv) extremely high porosities with commensurate high drug loading capacity and (v) the ability to tailor the size, shape and chemical nature of the internal and external surfaces thus allowing a high degree of control over drug-binding and release kinetics^19,20^. We have focussed our initial work on the iron-containing MOF, nanoMIL-89, since iron-MOFs have the added advantage of potentially being imageable using magnetic resonance imaging (MRI)^21,22^. Previous in vivo studies conducted using another iron-MOF from the same MIL family, MIL-100, showed its accumulation in the lung and gave signals detected by MRI, the accumulation was further confirmed using histological studies^23^. Other studies showed the in vitro detectability of another iron MOF from the MIL family,MIL-101 using MRI^24^ as well as other related MOFs such as nanoMIL-89 and MIL-53^25^.In addition to its potential detectability using MRI, nanoMIL-89 has a predicted cavity/pore size that is suitable for encapsulating PAH drugs^10^. In our hands, MIL-89 was shown to be relatively non-toxic in a range of human cell types, including endothelial and vascular smooth muscle cells, and was well tolerated in vivo for two weeks^10^. In line with this, others have found that a related iron-based MOF was well tolerated in vivo for up to three months^26^.

Here we have extended our feasibility studies by firstly preparing sildenafil-loaded nanoMIL-89 (Sil@nanoMIL-89) and then studying its drug release performance and corresponding vasodilator function.

## Results and discussion

### Chemical characterization of nanoMIL-89

**Figure 1:**
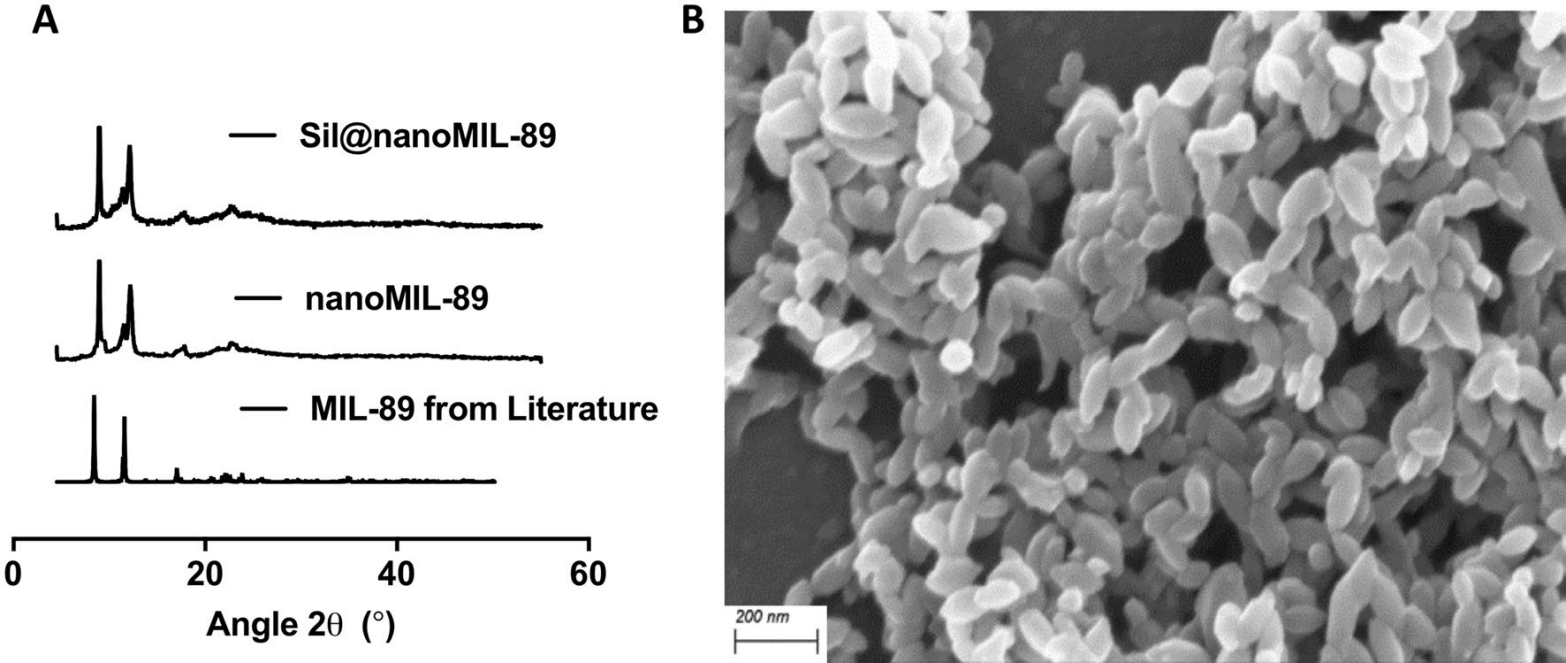
Characterization of nanoMIL-89. (**A**) Powder X-ray Diffraction (PXRD) analysis of nanoMIL-89 prepared within the reported study, MIL-89 reported in literature^20^ and nanoMIL-89 loaded with sildenafil (Sil@nanoMIL-89). (**B**) Scanning Electron Microscope image of nanoMIL-89 (× 5000).

NanoMIL-89 was prepared following previously reported procedures for MIL-89^10,25^ but with an additional step to include an optimised quantity of glacial acetic acid to the reaction mixture. Adjusting the amount of glacial acetic acid allowed control over the nanoparticle size and size distribution. The successful preparation of nanoMIL-89 was confirmed using powder X-ray diffraction (PXRD) analysis (Fig. 1A) and IR/ATR (Supplementary Fig. 1). Particle size was measured using dynamic light scattering (DLS) (Supplementary Fig. 2) and scanning electron microscopy (SEM) (Fig. 1B). SEM analysis of solid nanoMIL-89 revealed ovoid nanoparticles of length 82.5 ± 20.2 nm and width 31.4 ± 6.6 nm (n = 20 nanoparticles from 3 consecutive batches of nanoMIL-89) consistent with the 50–100 nm particle size reported in the original study by Horcajada et al*.*^25^. DLS analysis of 3 consecutive batches of nanoMIL-89 in solvent showed an estimated gyration diameter of 87.6 ± 25.3 nm (Supplementary Fig. 2), 137.1 ± 40.3 nm, and 147.6 ± 17.98 nm. These results confirm that nanoMIL-89 prepared in the current study was in the appropriate nanoscale range for biological applications (i.e., < 150 nm) and conforms to the expected composition and structure. This study aimed to extend our previous work^10^ and load nanoMIL-89 with a relevant PAH drug, namely sildenafil. The stability of the nanoMOF was confirmed by PXRD studies after suspending it overnight (16–18 h) in phosphate buffer saline (PBS; pH 7.4), which is the medium used for the drug loading studies, both in the absence and presence of sildenafil. Moreover, the PXRD pattern of nanoMIL-89 and Sil@nanoMIL-89 were consistent, indicating that the structure retained its integrity after loading (Fig. 1A).

### Sildenafil loading and release studies

**Figure 2:**
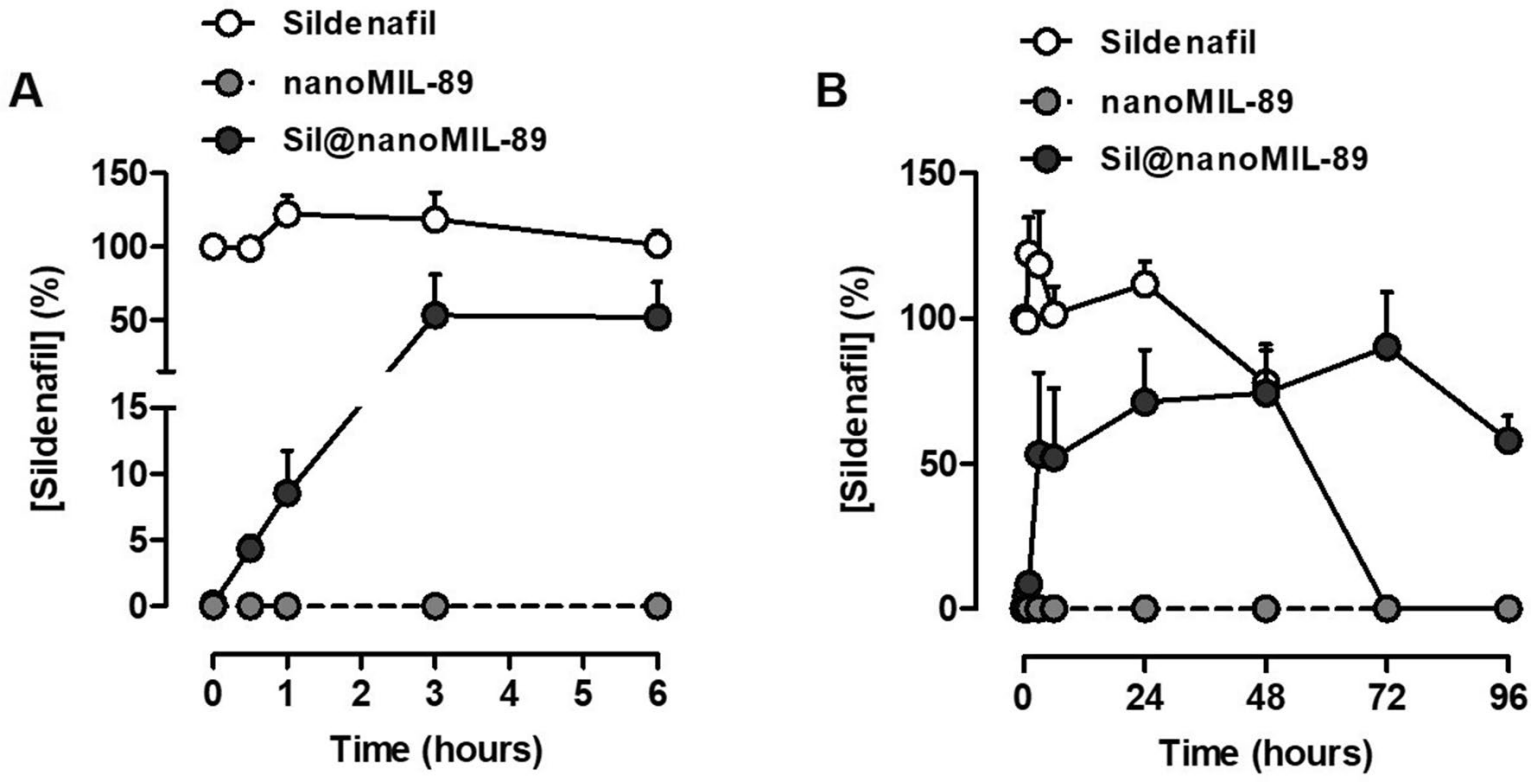
Sildenafil release by Sil@nanoMIL-89. Sildenafil levels in plasma were measured from incubations of Sil@nanoMIL-89 (4 mg/ml), nanoMIL-89 (4 mg/ml) and sildenafil (1 mg/ml) at 37 °C over (**A**) 6 h and (**B**) 96 h. Data are mean ± SEM for n = 6. Statistical significance was determined by two-way ANOVA followed by Tukey's multiple comparisons test. Statistical significance was assumed where *p < 0.05.

Loading studies showed that, under our experimental conditions, nanoMIL-89 readily absorbed > 90% of the sildenafil in the loading solution to yield Sil@nanoMIL-89. This, on a mass basis, amounted to 17% of the starting weight of nanoMIL-89. The high efficiency of sildenafil uptake from the loading solution and high loading weight percent (wt%) are both promising for potential medical applications. In addition, since the uptake of sildenafil from the loading solution was virtually complete, it is possible that even higher loading capacities can be achieved. Sildenafil release from Sil@nanoMIL-89 was then measured using human plasma as a matrix. As expected, plasma incubated with nanoMIL-89 did not react with reagents in the sildenafil ELISA (Fig. 2). Authentic sildenafil was relatively stable in plasma for up to 48 h, after which it was utterly degraded to undetectable levels. Sil@nanoMIL-89 exhibited an early steady release of sildenafil with detectable levels present in plasma within the first hour (Fig. 2A). After 6 h, Sil@nanoMIL-89 was still actively releasing the drug, although at a reduced rate (Fig. 2B). Interestingly, at the 72 and 96 h time points when free sildenafil (i.e., in the absence of the MOF) had degraded, the Sil@nanoMIL-89 sample continued to show high and consistent levels of sildenafil, presumably due to its continuing slow-release (Fig. 2B). The maximum amount of sildenafil released by Sil@nanoMIL-89 in plasma corresponds to 51% of the initially loaded drug, although the actual value could be higher due to the degradation of the sildenafil in plasma over time.

### Effect of nanoMIL-89 and Sil@nanoMIL-89 on cell viability

**Figure 3:**
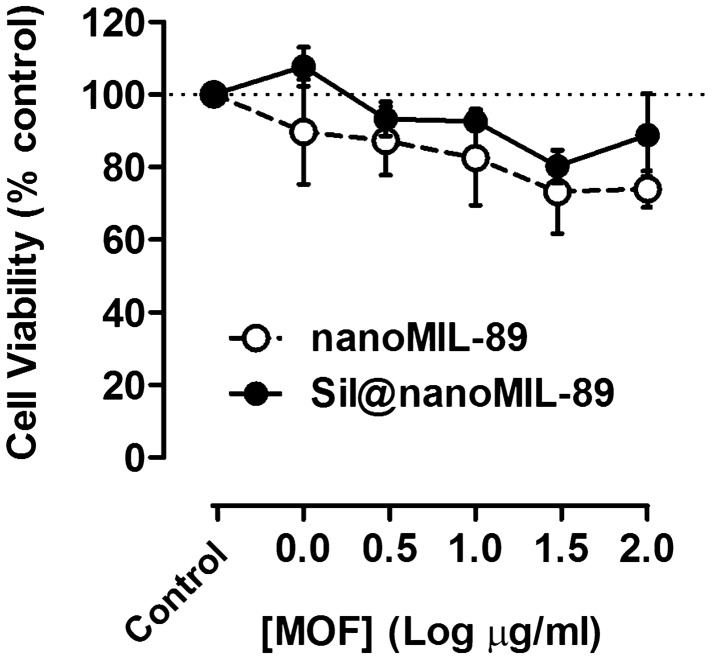
Effect of nanoMIL-89 and Sil@nanoMIL-89 on cell viability in human blood outgrowth endothelial cells. Data are shown as mean ± SEM for n = 8 determinations using cells from 4 separate isolations. Effect on viability was calculated at % control. Statistical analysis for effects between nanoMIL-89 and Sil@nanoMIL-89 was determined by two-way ANOVA followed by Bonferroni Multiple Comparison test where statistical significance was assumed where (^#^P < 0.05) and for each one compared to the relevant controls by one-way ANOVA followed by Dunnett’s Multiple Comparison Tests where statistical significance was assumed where (*P < 0.05). Analysis was performed on non-normalised data shown in Supplementary Fig. 3.

**Figure 4:**
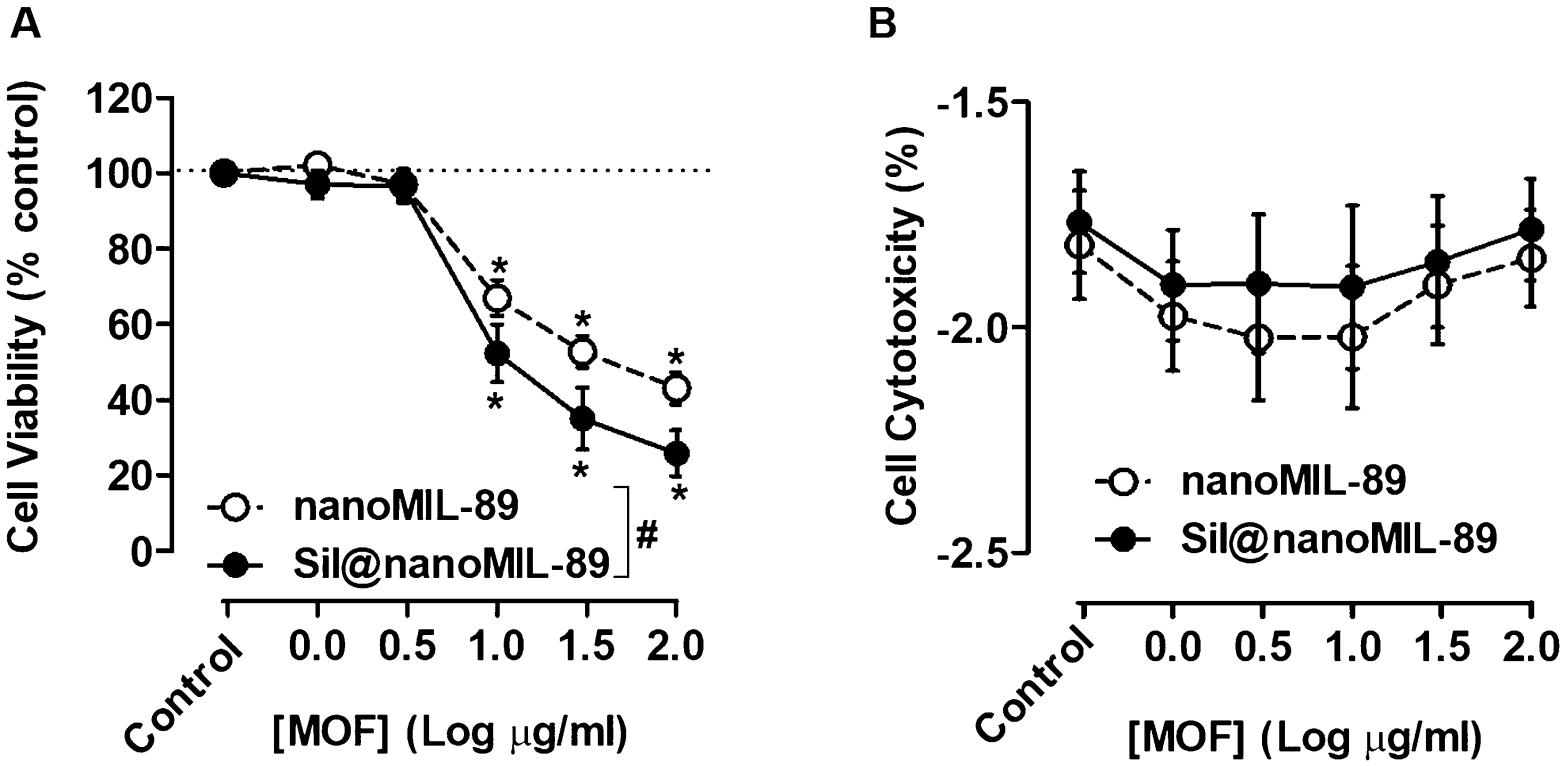
Effect of nanoMIL-89 and Sil@nanoMIL-89 on cell viability in human pulmonary artery smooth muscle cells (HPASMCs). Data are shown as mean ± SEM for n = 6 determinations using cells from 3 different donors. Effect on viability was calculated at % control for (**A**) and % cytotoxicity for (**B**). Statistical analysis for effects between nanoMIL-89 and Sil@nanoMIL-89 was determined by two-way ANOVA followed by Bonferroni Multiple Comparison test where statistical significance was assumed where (^#^P < 0.05) and for each one compared to the relevant controls by one-way ANOVA followed by Dunnett’s Multiple Comparison Tests where statistical significance was assumed where (*P < 0.05). Analysis was performed on non-normalised data shown in Supplementary Fig. 4.

As we have shown previously for nanoMIL-89^10^ in the current study, we found that despite a trend for reduction seen, neither nanoMIL-89 nor Sil@nanoMIL-89 showed statistically significant effects on the viability of human endothelial cells after 24 h incubation (Fig. 3 and Supplementary Fig. 3). In addition, as we have shown previously^10^, nanoMIL-89 reduced the viability of human pulmonary artery smooth muscle cells (HPASMCs). Sil@nanoMIL-89 also reduced HPASMCs viability and displayed a greater potency than was seen for nanoMIL-89 (Fig. 4A and Supplementary Fig. 4A). Neither MOF increased Lactate Dehydrogenas (LDH) release (Fig. 4B and Supplementary Fig. 4B), suggesting that reduced viability was associated with inhibition of proliferation rather than cell death. These effects may be considered as therapeutically important in treating the remodelling associated with PAH.

### Vasodilator responses of Sil-nanoMIL-89

**Figure 5:**
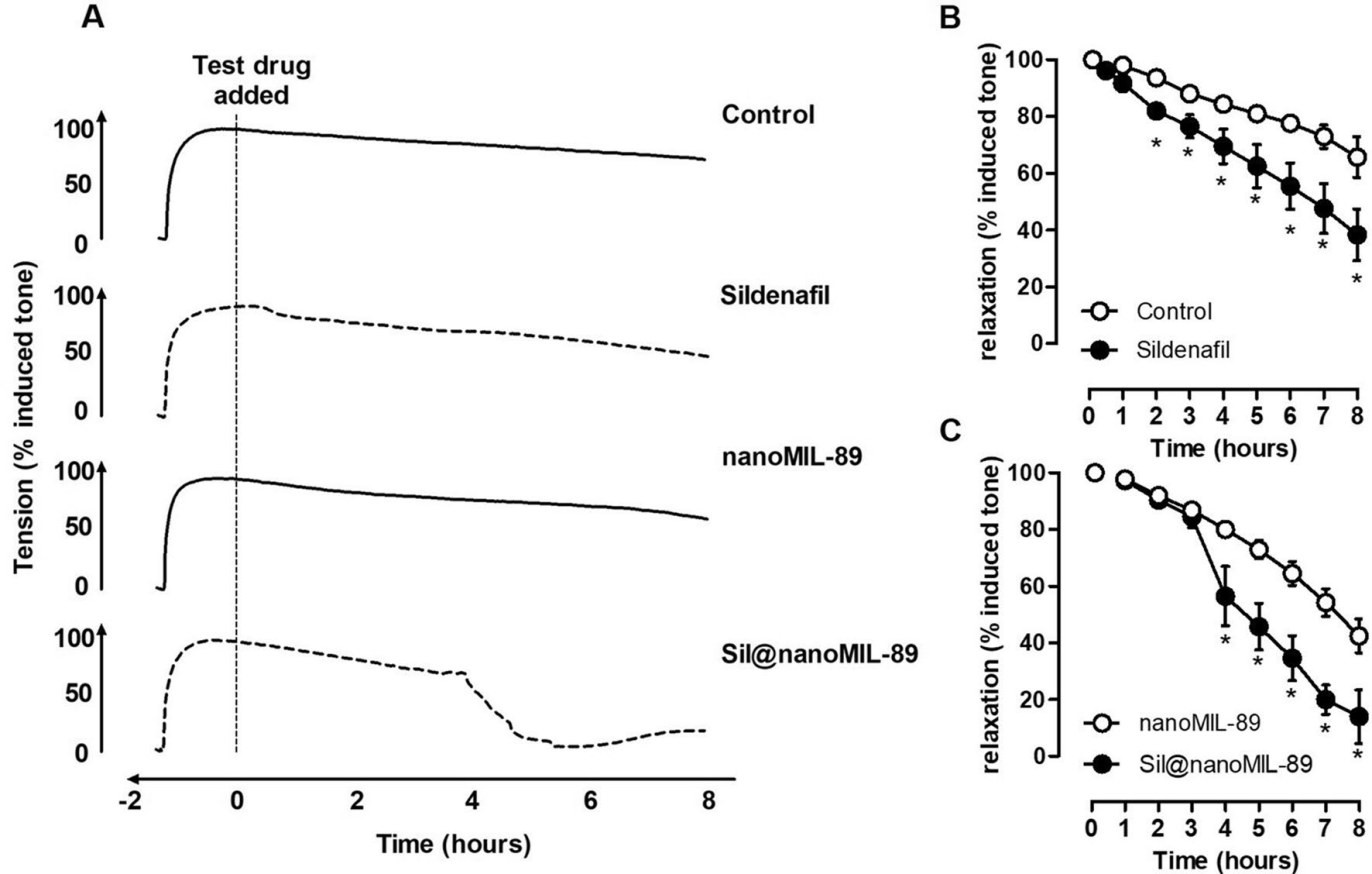
Vasodilator effects of sildenafil (10 µM), nanoMIL-89 (10 µg/ml), and Sil@nanoMIL-89 (10 µg/ml) on pre-contracted mouse aorta. The figure shows representative vessel responses across the full-time course (**A**) and pooled data presented as mean ± SEM for n = 3–8 vessels from 3–4 mice (**B**, **C**). Statistical significance was determined by two-way ANOVA followed by Tukey's multiple comparisons test. Statistical significance was assumed where *P < 0.05.

Over the course of 8 h, mouse aorta contracted with U46619, the synthetic mimetic of the endoperoxide prostaglandin PGH_2,_ which acts on thromboxane receptors in smooth muscle cells, retained approximately 70% of induced tone (Fig. 5). Sildenafil is a vasodilator drug that relaxes blood vessels by increasing the biological half-life of the cGMP. As expected, therefore, sildenafil induced vasodilation within the first 60 min and continued to relax vessels (compared to control) for the duration of the experiment (Fig. 5B). NanoMIL-89 also induced relaxation, but with a different time course, relaxation induced by nanoMIL-89 was not apparent (compared to PSS alone) until 4 h after the addition of the MOF (Fig. 5C). It is not clear why nanoMIL-89 induced relaxation, but one explanation might be that due to the high porosity of the nanoparticle, it is either absorbing the contractile agent U46619 or any of the other critical elements in the physiological solution that are required for the contraction. In line with sildenafil release data and considering the kinetics of vasorelaxation induced by sildenafil, Sil@nanoMIL-89 induced significant and sustained vasodilator response, which began after a lag phase of > 2–4 h and was sustained for the duration of the experiment (Fig. 5A,C). The time course of relaxation induced by Sil@nanoMIL-89 is inline with sildenafil release from the MOF (Fig. 2), when taking into account the kinetics of vasorelaxation induced by authentic sildenafil.The maximal relaxant effect of Sil@nanoMIL-89 (10 µg/ml) was significantly greater than the nanoMIL-89 (10 µg/ml) (Fig. 5).

## Conclusion

Here we have prepared, for the first time, a sildenafil-loaded nanoMOF material and assessed its potential in drug delivery applications. Sil@nanoMIL-89 retained its crystallinity, released sildenafil over a prolonged period of time, and induced vasodilation in a sustained manner. These results are consistent with the idea that nanoMIL-89 is a promising formulation prototype for PAH drugs and describe our first prototype drug (Sil@nanoMIL-89). Our next steps in developing these Sil@MOF drugs will be (i) to assess the pharmacology, toxicology, efficacy, and in vivo distribution of Sil@nanoMIL-89 and (ii) establish strategies for targeted delivery of Sil@nanoMIL-89 and related drugs to the pulmonary vasculature.

## Material and methods

### Preparation of nanoMIL-89

NanoMIL-89 was prepared following a modification of the previously published procedure by Horcajada et al.^25^, with the addition of an optimised quantity of glacial acetic acid to the reaction mixture to control particle size and quality. In brief, 10 mmol of iron(III) chloride hexahydrate (FeCl_3_·6H_2_O; Sigma Aldrich, UK) and 10 mmol *trans*, *trans*-muconic acid (Sigma Aldrich, UK) were mixed in absolute ethanol (100 ml; 99.8%; VWR, UK). The reaction mixture was then sonicated for 15 min, and 20 ml of glacial acetic acid (99.8%; VWR, UK) was added. The mixture was heated at 90 °C for 24 h in a Parr reactor, after which the precipitate was recovered by centrifugation at 7000 rpm for 15 min. The precipitate was washed with distilled water and vacuum dried to recover the nanoMIL-89 as a brown precipitate (100–200 mg/reaction).

### Chemical characterization of nanoMIL-89

The successful preparation of nanoMIL-89 was confirmed using powder X-ray diffraction (PXRD; Bruker D2 Phaser) and infrared/attenuated total reflection (IR/ATR; Perkin Elmer Spectrum). Particle size was measured using dynamic light scattering (DLS; DelsaNano C by Beckman Coulter), where 50 μl of the reaction mixture was taken and added to 5 ml of ethanol, which was then centrifuged for 2 min before 100 μl of the supernatant was used for DLS analysis. In addition, particle size was estimated from images obtained from dried nanoMIL-89 samples using scanning electron microscopy (SEM; LEO Gemini 1525 by Zeiss), analysed and quantified using ImageJ Launcher Software^27^ to identify the particulate size. Briefly, 20 nanoparticles were selected randomly, with the only prerequisite being that they are well defined. Length and width measurements were made using ImageJ. PXRD analysis was consistent with literature patterns for bulk MIL-89 (see Fig. 1)^10,28,29^.

### Loading and release studies of nanoMIL-89 with sildenafil

The sildenafil nanoMIL-89 loading procedure, to produce Sil@nanoMIL-89, was conducted as follows: 5 ml of a 1 mg/ml sildenafil solution was prepared in phosphate buffer saline (PBS; pH 7.4; Sigma Aldrich, UK), then 20 mg of nanoMIL-89 was added to the solution and incubated on a shaker at room temperature for 16–18 h. The concentration of sildenafil was selected to be just below its maximum solubility in aqueous solutions. The resultant Sil@nanoMIL-89 precipitate was retrieved by centrifugation at 7000 rpm at room temperature for 15 min. Samples of the supernatant were collected to assess levels of sildenafil remaining after loading and were used to calculate the amount of drug taken up by the nanoMIL-89. The precipitated Sil@nanoMIL-89 was then re-suspended in 5 ml of human plasma from 3 separate donors; Human plasma samples were purchased from Cambridge Bioscience Company, UK. Solutions of Sil@nanoMIL-89 were incubated at 37 °C for 0, 0.5, 1, 3, 6, 16, 24, 36, 48, 60, 72, 84 and 96 h. At these time points, the solution was centrifuged at 7000 rpm at room temperature for 15 min. The release of sildenafil into the supernatant was measured at each time point by ELISA (MaxSignal; Sildenafil/Vardenafil ELISA Test Kit (MEDIBENA, UK) following the manufacturer’s instructions.

### Human endothelial cell viability response to Sil@nanoMIL-89

Human blood outgrowth endothelial cells were isolated, cultured, plated, and treated as described previously^10^. Cell viability was indicated by changes in respiration using alamarBlue reagent according to the manufacturer’s instructions.

### Human pulmonary artery smooth muscle cell viability response to Sil@nanoMIL-89

Human pulmonary artery smooth muscle cells were purchased from Promocell. Cells were then cultured, plated, and treated as described previously^10^. Cell viability was monitored by changes in respiration using alamarBlue reagent according to the manufacturer’s instructions. The effects of drugs on cell cytotoxicity was measured using LDH (Abcam, UK), following the manufacturer’s instructions.

### Aorta vasomotor responses to Sil@nanoMIL-89

C57 Black 4 mice (6–10 weeks) were killed by CO_2_ narcosis and aorta removed, cleaned of connective tissue and cut into 1.5 mm rings before being mounted in Mulvany-Halpern myograph organ baths containing a physiological salt solution (PSS), as we have described previously^30^. In order to optimize the stability of vascular function over the 8-h time course, diclofenac (1 µM) and cycloheximide (1 µM) were added to the PSS to block vasoactive prostanoids and induction of vasoactive genes (e.g., NO synthase) respectively. Vessels were contracted with an EC_80_ concentration of U46619 (10 nM). Once a stable baseline was obtained, sildenafil (10 µM), nanoMIL-89 (10 µg/ml), or Sil@nanoMIL-89 (10 µg/ml) was added to the PSS, and vascular tone monitored for 8 h. Responses in vessels incubated in PSS served as controls. Force was recorded via a PowerLab/800 (AD Instruments Ltd., UK) and analysed using Chart 6.0 acquisition system (AD Instruments Ltd., UK). All studies using animals or animal tissues/organs were conducted in accordance with UK Home Office Animals (Scientific Procedures) Act 1986.

### Methods and experimental protocols

All methods and experimental protocols were performed in accordance with the relevant guidelines and regulations approved by Imperial College London. In addition, the study protocols were approved by the ethics committee at Imperial College of London.

### Statistical analysis

Data were presented as mean ± SEM, and statistical significance (taken as P < 0.05) was determined using GraphPad Prism 7 as described in each Figure legend.

## Supplementary Information

Supplementary Information.

## Data Availability

The dataset analysed during the current study are available from the corresponding author on reasonable request.

## Abbreviations

DLS: Dynamic light scattering
ELISA: Enzyme-linked immunosorbent assay
FeCl_3_·6H_2_O: Iron(III) chloride hexahydrate
HPASMCs: Human pulmonary artery smooth muscle cells
IR/ATR: Infrared/attenuated total reflection
LDH: Lactate dehydrogenase
MOF: Metal–organic framework
NO: Nitric oxide
PAH: Pulmonary arterial hypertension
PBS: Phosphate buffer saline
PSS: Physiological salt solution
PXRD: Powder X-ray diffraction
SEM: Scanning electron microscopy
Sil@nanoMIL-89: Sildenafil loaded nanoMIL-89

## References

[1] Hemnes AR, Champion HC (2006). Sildenafil, a PDE5 inhibitor, in the treatment of pulmonary hypertension. Expert Rev. Cardiovasc. Ther..

[2] Larche NE, Mousa SA (2013). Riociguat for the management of pulmonary arterial hypertension and chronic thromboembolic pulmonary hypertension. Drugs Today.

[3] Miyauchi T, Masaki T (1999). Pathophysiology of endothelin in the cardiovascular system. Annu. Rev. Physiol..

[4] Roberts KE, Preston IR (2009). Safety and tolerability of bosentan in the management of pulmonary arterial hypertension. Drug. Des. Dev. Ther..

[5] McLaughlin VV, Shillington A, Rich S (2002). Survival in primary pulmonary hypertension: The impact of epoprostenol therapy. Circulation.

[6] Lindegaard Pedersen M, Kruger M, Grimm D, Infanger M, Wehland M (2019). The prostacyclin analogue treprostinil in the treatment of pulmonary arterial hypertension. Basic Clin. Pharmacol. Toxicol..

[7] Del Pozo R, Hernandez Gonzalez I, Escribano-Subias P (2017). The prostacyclin pathway in pulmonary arterial hypertension: A clinical review. Expert Rev. Respir. Med..

[8] Kuwano K, Hashino A, Noda K, Kosugi K, Kuwabara K (2008). A long-acting and highly selective prostacyclin receptor agonist prodrug, 2-{4-[(5,6-diphenylpyrazin-2-yl)(isopropyl)amino]butoxy}-N-(methylsulfonyl)acetam ide (NS-304), ameliorates rat pulmonary hypertension with unique relaxant responses of its active form, {4-[(5,6-diphenylpyrazin-2-yl)(isopropyl)amino]butoxy}acetic acid (MRE-269), on rat pulmonary artery. J. Pharmacol. Exp. Ther..

[9] Mitchell JA (2014). Role of prostacyclin in pulmonary hypertension. Glob. Cardiol. Sci. Pract..

[10] Mohamed NA (2017). Chemical and biological assessment of metal organic frameworks (MOFs) in pulmonary cells and in an acute in vivo model: relevance to pulmonary arterial hypertension therapy. Pulm. Circ..

[11] Mohamed NA (2016). A new NO-releasing nanoformulation for the treatment of pulmonary arterial hypertension. J. Cardiovasc. Transl. Res..

[12] Segura-Ibarra V (2018). Nanotherapeutics for treatment of pulmonary arterial hypertension. Front. Physiol..

[13] Brenner JS, Greineder C, Shuvaev V, Muzykantov V (2015). Endothelial nanomedicine for the treatment of pulmonary disease. Expert Opin. Drug Deliv..

[14] Mosgoeller W, Prassl R, Zimmer A (2012). Nanoparticle-mediated treatment of pulmonary arterial hypertension. Methods Enzymol..

[15] Rashid J, Nahar K, Raut S, Keshavarz A, Ahsan F (2018). Fasudil and DETA NONOate, loaded in a peptide-modified liposomal carrier, slow PAH progression upon pulmonary delivery. Mol. Pharm..

[16] Jain PP (2014). Liposomal nanoparticles encapsulating iloprost exhibit enhanced vasodilation in pulmonary arteries. Int. J. Nanomed..

[17] Akbarzadeh A (2013). Liposome: Classification, preparation, and applications. Nanoscale Res. Lett..

[18] Erik Brewer JC, Lowman A (2011). Emerging technologies of polymeric nanoparticles in cancer drug delivery. J. Nanomater..

[19] Kızılel SKAS (2011). Biomedical applications of metal organic frameworks. Ind. Eng. Chem. Res..

[20] Horcajada P (2006). Metal-organic frameworks as efficient materials for drug delivery. Angew. Chem..

[21] Estelrich J, Sanchez-Martin MJ, Busquets MA (2015). Nanoparticles in magnetic resonance imaging: From simple to dual contrast agents. Int. J. Nanomed..

[22] Prince MR, Zhang HL, Chabra SG, Jacobs P, Wang Y (2003). A pilot investigation of new superparamagnetic iron oxide (ferumoxytol) as a contrast agent for cardiovascular MRI. J. Xray Sci. Technol..

[23] Wang D (2016). Controllable synthesis of dual-MOFs nanostructures for pH-responsive artemisinin delivery, magnetic resonance and optical dual-model imaging-guided chemo/photothermal combinational cancer therapy. Biomaterials.

[24] Wyszogrodzka G (2018). Iron-based metal-organic frameworks as a theranostic carrier for local tuberculosis therapy. Pharm. Res..

[25] Horcajada P (2010). Porous metal-organic-framework nanoscale carriers as a potential platform for drug delivery and imaging. Nat. Mater..

[26] Simagina, A. A. Vol. 9 (ed. M. V. Polynski) (Russian Academy of Sciences and Turpion Ltd, Russia, 2018).

[27] Schneider CA, Rasband WS, Eliceiri KW (2012). NIH Image to ImageJ: 25 years of image analysis. Nat. Methods.

[28] Serre C, Surble S, Mellot-Draznieks C, Filinchuk Y, Ferey G (2008). Evidence of flexibility in the nanoporous iron(iii) carboxylate MIL-89. Dalton Trans..

[29] Surble S, Millange F, Serre C, Ferey G, Walton RI (2006). An EXAFS study of the formation of a nanoporous metal-organic framework: Evidence for the retention of secondary building units during synthesis. Chem. Commun..

[30] Ahmetaj-Shala B (2015). Evidence that links loss of cyclooxygenase-2 with increased asymmetric dimethylarginine: Novel explanation of cardiovascular side effects associated with anti-inflammatory drugs. Circulation.

