# Supplementary material for: Studies on metal–organic framework (MOF) nanomedicine preparations of sildenafil for the future treatment of pulmonary arterial hypertension
Source: Sci Rep. 2021 Feb 22;11:4336. doi: 10.1038/s41598-021-83423-6 (PMC7900107; doi:10.1038/s41598-021-83423-6)

Studies on Metal-organic framework (MOF) nanomedicine preparations of sildenafil for the future treatment of pulmonary arterial hypertension

*Nura A. Mohamed[a], *Haissam Abou-Saleh[a,g], Yu Kameno[b], Isra Marei[c,d], Gilberto de Nucci[e,f], Blerina Ahmetaj-Shala[c], Fisnik Shala[c], Nicholas S.Kirkby[c], Lewis Jennings[b], Dana E. Al-Ansari[a], Robert P. Davies[b], Paul D. Lickiss[b,] and Jane A. Mitchell[c]

[a]Department of Biological and Environmental Sciences, College of Arts and Sciences, Qatar University, Doha, Qatar.

[b]Department of Chemistry, White City Campus, Imperial College, London, UK.

[c]Department of Cardiothoracic Pharmacology, National Heart and Lung Institute, Imperial College, London, UK.

[d]Qatar Foundation Research and Development Division, Doha, Qatar.

[e]Department of Pharmacology, Faculty of Medical Sciences, State University of Campinas (UNICAMP), Campinas, SP, Brazil.

[f]Department of Pharmacology, Institute of Biomedical Sciences, University of São Paulo, São Paulo, SP, Brazil.

[g]Biomedical Research Center, Qatar University, Doha, Qatar.

Supplementary Figure 1: Infrared/attenuated total reflection (IR/ATR) spectra for nanoMIL-89. The figure shows a representative IR/ATR tracing.

Supplementary Figure 2: Estimation of nanoMIL-89 size using dynamic light scattering (DLS) analysis.

Supplementary Figure 3: Effect of nanoMIL-89 and Sil@nanoMIL-89 on cell viability in human blood outgrowth endothelial cells. Data are shown as mean ± SEM for n=8 determinations using cells from 4 separate isolations. Effect on viability is represented as absorbance 570-620 values. Statistical analysis for effects between nanoMIL-89 and Sil@nanoMIL-89 was determined by two-way ANOVA followed by Bonferroni Multiple Comparison test where statistical significance was assumed where (#$P < 0.05$) and for each one compared to the relevant controls by one-way ANOVA followed by Dunnett's Multiple Comparison Tests where statistical significance was assumed where (*$P < 0.05$).

Supplementary Figure 4: Effect of nanoMIL-89 and Sil@nanoMIL-89 on cell viability in human pulmonary artery smooth muscle cells (HPASMCs). Data are shown as mean ± SEM for n=6 determinations using cells from 3 different donors. Values are represented as absorbance 570-620 for A and absorbance 450 for B. Statistical analysis for effects between nanoMIL-89 and Sil@nanoMIL-89 was determined by two-way ANOVA followed by Bonferroni Multiple Comparison test where statistical significance was assumed where (#$P < 0.05$) and for each one compared to the relevant controls by one-way ANOVA followed by Dunnett's Multiple Comparison Tests where statistical significance was assumed where (*$P < 0.05$).

Supplementary Figure 1

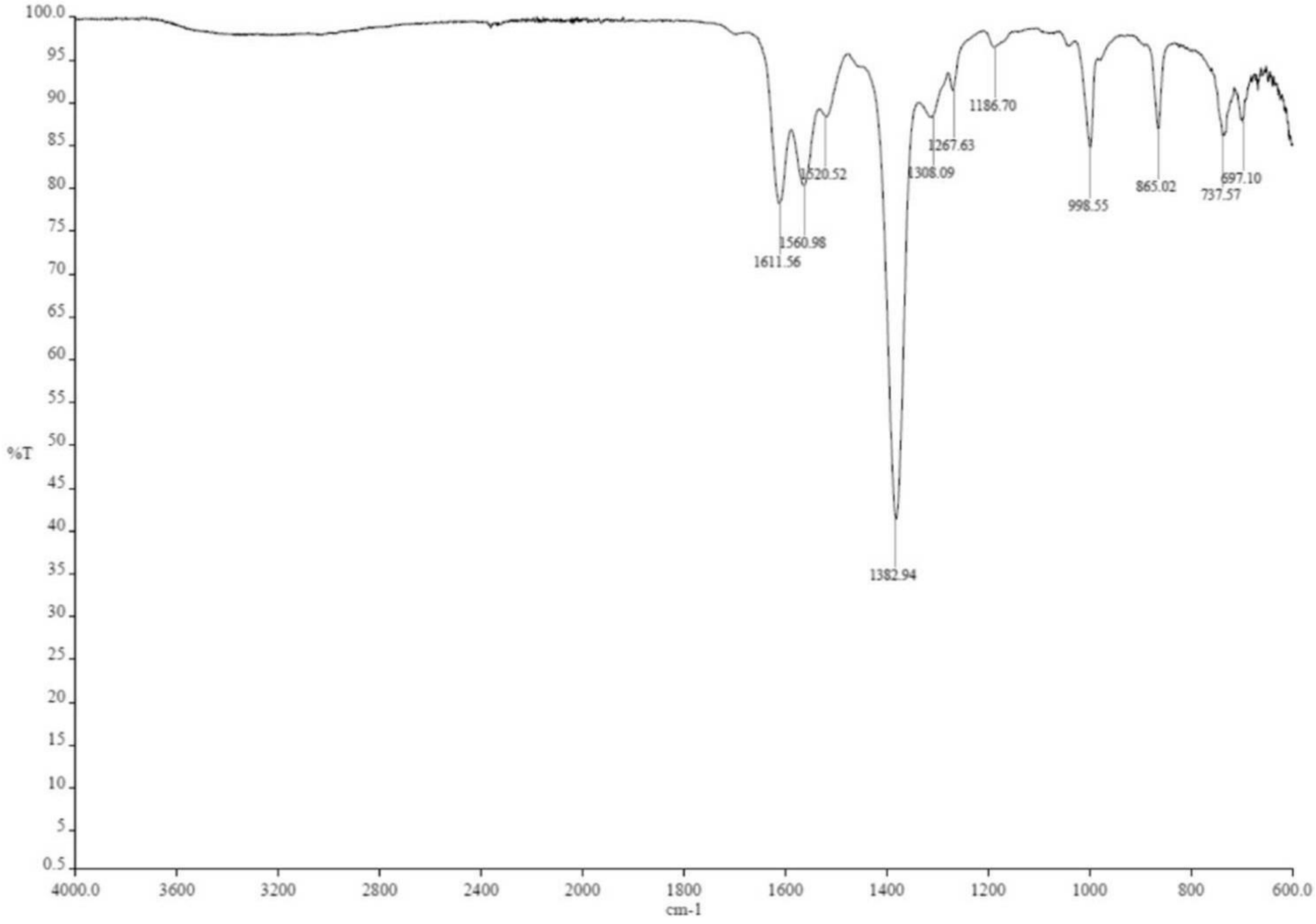

Supplementary Figure 2

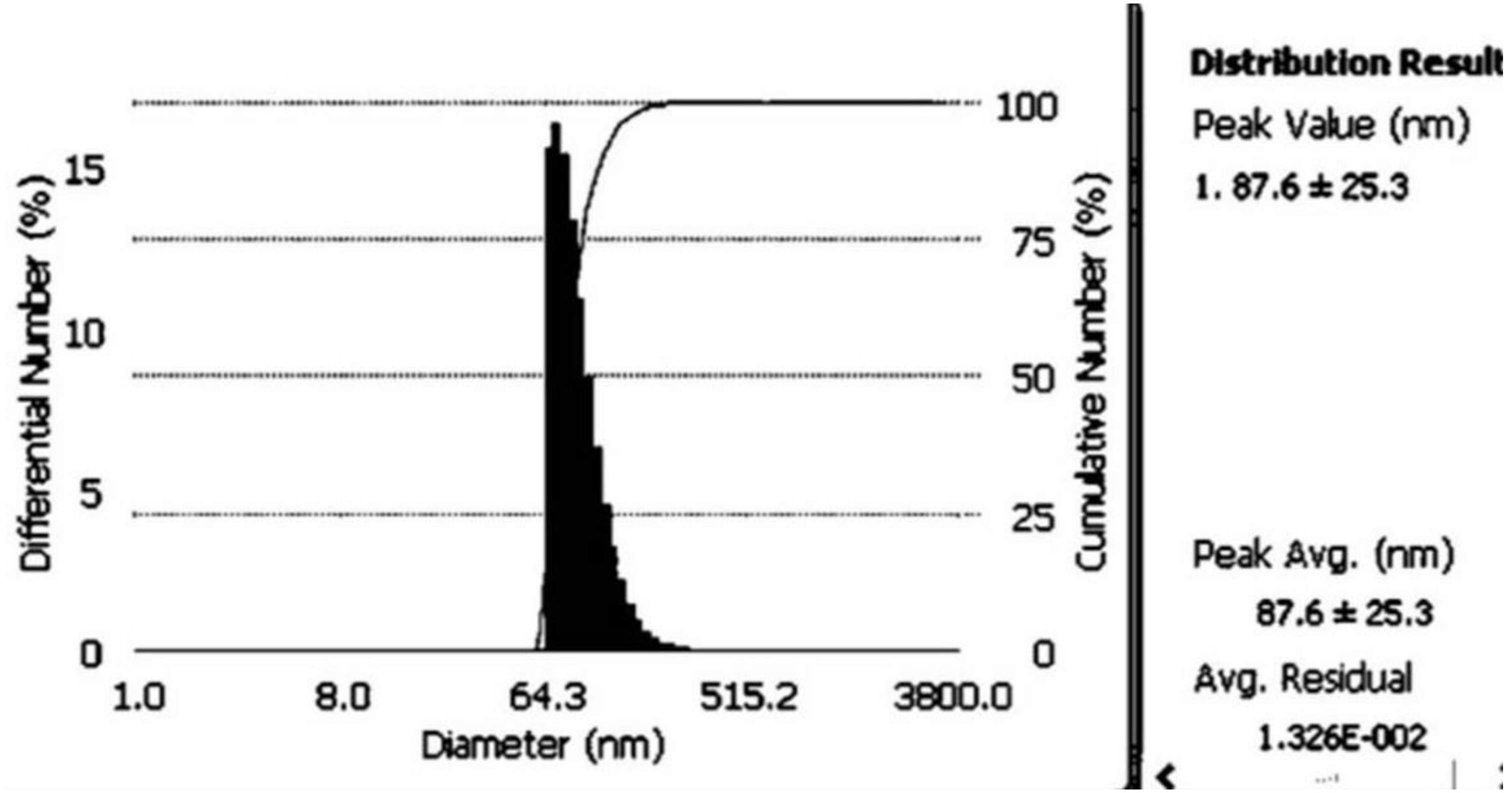

Supplementary Figure 3

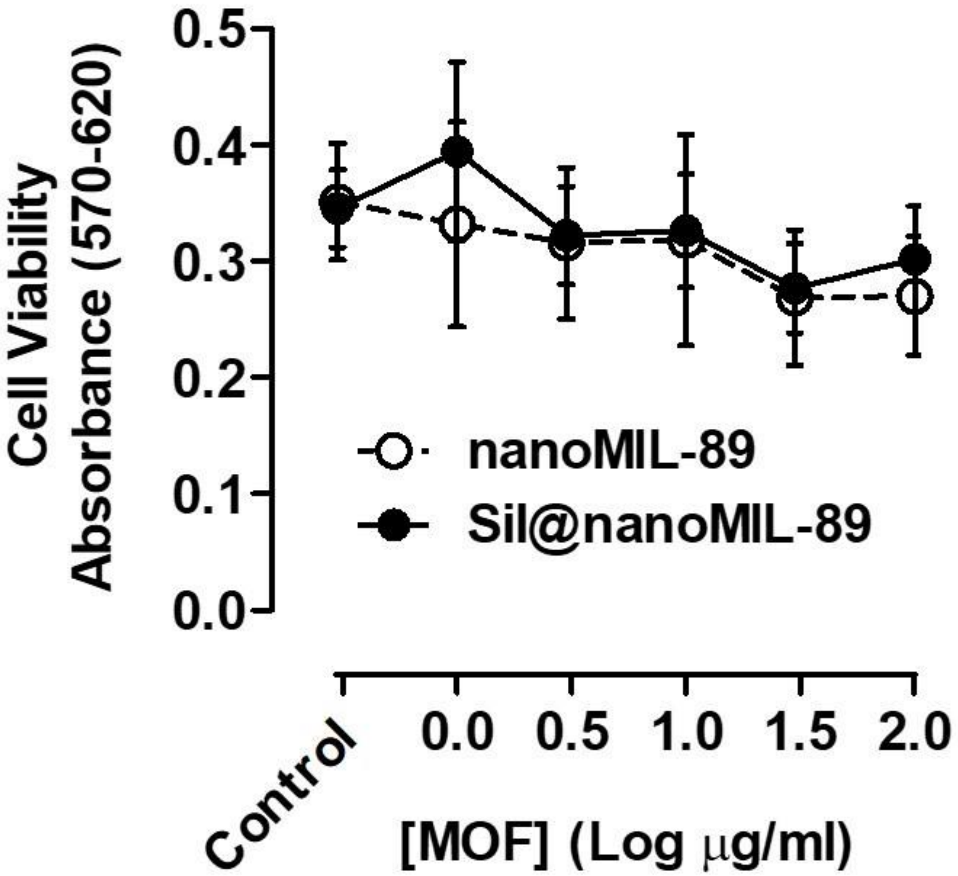

Supplementary Figure 4

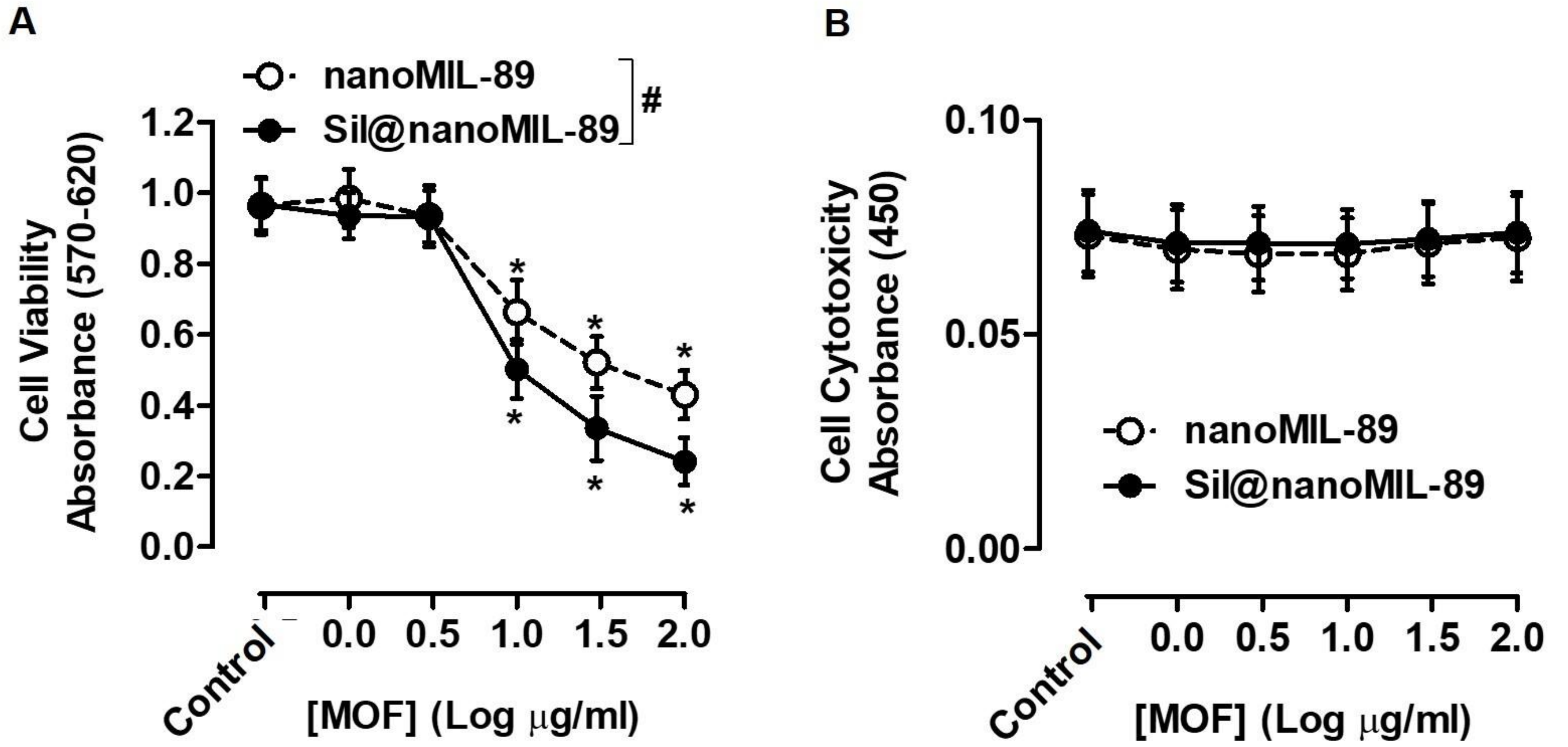

Supplement: Supplementary file 1 — Supplementary Information. [file 41598_2021_83423_MOESM1_ESM.pdf]
